# Supplementary material for: Impacts of Long COVID on workers: A longitudinal study of employment exit, work hours and mental health in the UK
Source: PLoS One. 2024 Jun 26;19(6):e0306122. doi: 10.1371/journal.pone.0306122 (PMC11207153; doi:10.1371/journal.pone.0306122)
Supplement: S1 Appendix — (DOCX) [file pone.0306122.s001.docx]

**Supplementary Document: S1 Appendix**

S1 Table 1. Sample description, wave-observations

| Variables | Sample 1: employed in Jan/Feb 2020 | Sample 2: employed in Jan/Feb 2020 and at *t* (in wave 7, 8 or 9) |
| --- | --- | --- |
| COVID-19 symptoms at *t* | (n=22,106) | (n=20,225) |
| No COVID-19 symptoms (reference) | 89.7% | 89.5% |
| Symptoms for <5 weeks | 7.8% | 8.1% |
| Long COVID for 5-28 weeks | 1.6% | 1.6% |
| Long COVID for 29+ weeks | 0.9% | 0.9% |
| Employed at *t* | (n=22,106) | (n=20,225) |
| Yes | 91.5% | 100% |
| No | 8.5% | 0% |
| Sex | (n=22,090) | (n=20,212) |
| Male | 41.2% | 41.5% |
| Female | 58.8% | 58.6% |
| Lives with partner/spouse at *t* | (n=22,106) | (n=20,225) |
| Yes | 72.5% | 73.1% |
| No | 27.5% | 26.9% |
| Lives with child 0-15 years old at *t* | (n=22,106) | (n=20,225) |
| Yes | 32.2% | 33.6% |
| No | 67.8% | 66.4% |
| Ethnicity (from 2019) | (n=21,890) | (n=20,025) |
| White British | 84.1% | 84.2% |
| White, other | 4.8% | 4.8% |
| Mixed | 1.9% | 2.0% |
| Asian or Asian British | 6.5% | 6.4% |
| Black or Black British | 2.3% | 2.2% |
| Other ethnic group | 0.5% | 0.5% |
| Mean age at *t* | 48.03, sd=12.8 (n=22,106) | 47.48, sd=12.4 (n=20,225) |
| Mean hours worked in Jan/Feb 2020 | 33.08, sd=13.0 (n=22,015) | 33.82, sd=12.5 (n=20,148) |
| Mean net earnings per hour (£) in Jan/Feb 2020 | 14.24, sd=18.6 (n=20,070) | 14.28, sd=19.0 (n=18,438) |
| Was temporarily employed in 2019 | (n=21,112) | (n=19,325) |
| Yes | 8.7% | 8.2% |
| No | 91.3% | 91.8% |
| Was self-employed in 2019 | (n=22,106) | (n=20,225) |
| Yes | 12.5% | 12.1% |
| No | 87.5% | 87.9% |
| Industry in 2019 (SIC 2007) | (n=19,194) | (n=17,744) |
| SIC AB | 0.7% | 0.7% |
| SIC CDEF | 12.9% | 13.1% |
| SIC GI | 14.1% | 13.8% |
| SIC HJ | 8.4% | 8.3% |
| SIC KLMN | 16.7% | 16.9% |
| SIC OPQ | 42.4% | 42.4% |
| SIC RSTU | 4.9% | 4.8% |
| Long-standing illness or disability in 2019 | (n=21,098) | (n=19,310) |
| Yes | 28.2% | 27.3% |
| No | 71.8% | 72.7% |
| Highest qualification (in 2019) | (n=20,986) | (n=19,207) |
| low | 6.2% | 5.9% |
| medium | 37.0% | 36.7% |
| high | 56.7% | 57.4% |
| Region of residence at *t* | (n=21,594) | (n=19,751) |
| North East | 3.3% | 3.5% |
| North West | 9.6% | 9.6% |
| Yorkshire and Humble | 8.3% | 8.3% |
| East Midlands | 7.9% | 7.9% |
| West Midlands | 8.4% | 8.5% |
| East of England | 10.2% | 10.2% |
| London | 9.9% | 9.9% |
| South East | 14.0% | 13.8% |
| South West | 9.6% | 9.6% |
| Wales | 5.6% | 5.6% |
| Scotland | 8.8% | 8.9% |
| Northern Ireland | 4.4% | 4.5% |
| Self-employed at *t* |  | (n=20,225) |
| Yes |  | 12.4% |
| No |  | 87.6% |
| Industry at *t* (SIC 2007) |  | (n=20,069) |
| SIC AB |  | 1.4% |
| SIC CDEF | | 12.3% |
| SIC GI |  | 12.4% |
| SIC HJ | | 8.2% |
| SIC KLMN | | 16.6% |
| SIC OPQ | | 36.5% |
| SIC RSTU |  | 12.6% |
| Mean net earnings per hour (£) at *t* |  | 15.86, sd=23.3 (n=15,559) |
| Mean reversed GHQ-12 at *t* |  | 23.62, sd=5.8 (n=19,659) |
| Mean happiness score at *t* |  | 3.01, sd=0.8 (n=19,729) |
| Works zero hours at *t* |  | 9.5% (n=19,979) |
| Mean hours worked at *t* |  | 34.4, sd=13.0 (n=18,073) |

*Source: Understanding Society COVID-19 Study 2020-2021, waves 7-9.*

*Note: unweighted data; t refers to the survey wave (7-9).*

*^1^AB- agriculture, mining; CDEF-manufacturing, electricity, gas, water, construction; GI-retail, wholesale, hospitality; HJ-transport, information and communication; KLMN-finance, insurance, professional & admin. Services; OPQ-public admin., health, social, education; RSTU-other personal services.*

S1 Table 2. Change in number of working hours between COVID-19 surveys (Jan-Sep 2021) and Jan/Feb 2020, fixed-effects (FE) and random-effects (RE) panel models

| Co-variates | FE Model | | RE Model | |
| --- | --- | --- | --- | --- |
|  | Coeff. | SE | Coeff. | SE |
| COVID-19 symptoms (Ref. none) |  |  |  | |
| <5 weeks | -1.456*** | 0.357 | -1.526*** | 0.321 |
| 5-28 weeks | -2.489** | 0.837 | -2.470** | 0.723 |
| 29+ weeks | 2.149 | 1.255 | 0.516 | 1.000 |
| Age | 1.683* | 0.734 | 0.455*** | 0.072 |
| Age^2^ | -0.021** | 0.007 | -0.006*** | 0.0008 |
| Child 0-15 | -1.728** | 0.667 | -0.698* | 0.278 |
| No partner (Ref. couple) | -0.349 | 0.599 | 0.839** | 0.279 |
| Industry (Ref. SIC-KLMN)^1,2^ |  |  |  | |
| SIC CDEF | 1.691 | 2.646 | 0.484 | 0.471 |
| SIC GI | -8.557*** | 2.294 | -4.757*** | 0.472 |
| SIC HJ | -2.143 | 2.770 | -1.274* | 0.522 |
| SIC OPQ | 4.083 | 2.129 | -0.062 | 0.364 |
| SIC RSTU | 2.458 | 2.539 | -2.473*** | 0.464 |
| Self-employed (yes) | -6.305*** | 1.482 | -4.627*** | 0.399 |
| Female (Ref. male) | - |  | -1.717*** | 0.283 |
| Ethnicity (Ref. White British)^1^ |  |  |  | |
| White, other | - |  | -0.376 | 0.603 |
| Asian or Asian British | - |  | -0.945 | 0.545 |
| Black or Black British | - |  | -0.891 | 0.857 |
| Highest qualification (Ref. high) |  |  |  | |
| Medium | - |  | -0.482 | 0.278 |
| Low | - |  | -0.285 | 0.570 |
| *Pre-pandemic characteristics (2019/2020)* |  |  |  | |
| Long-standing health cond. (Ref. no) | - |  | -0.888** | 0.280 |
| Net earnings per hour (logged) | - |  | 1.926*** | 0.240 |
| Constant | -31.309 | 19.527 | -3.444 | 1.805 |
| Wave-obs. | 19,801 |  | 16,622 | |
| Individuals | 8,524 |  | 7,213 | |
| F/Wald Chi2(df) | 16.02 |  | 1270.54(38) | |

*Source: USoc COVID-19 Study 2020-2021. Sample of respondents who were in work in Jan/Feb 2020 and are still in work at t (waves 7-9). Wave dummies are included and region dummies in the RE models.
 Significance level: ***p<0.001, **p<0.01, *p<0.05.*

*^1^Categories with small cell sizes not shown.*

*^2^CDEF-manufacturing, electricity, gas, water, construction; GI-retail, wholesale, hospitality; HJ-transport, information and communication; KLMN-finance, insurance, professional & admin. Services; OPQ-public admin., health, social, education; RSTU-other personal services.*
